# Geriatric Nutritional Risk Index as a Prognostic Factor for Renal Progression in Patients with Type 2 Diabetes Mellitus

**DOI:** 10.3390/nu15214636

**Published:** 2023-10-31

**Authors:** Eun Jung Kim, Ajin Cho, Do Hyoung Kim, Hayne Cho Park, Joo Yeon Yoon, Kyungjun Shon, Eunji Kim, Ja-Ryong Koo, Young-Ki Lee

**Affiliations:** 1 Department of Internal Medicine, Dongtan Sacred Heart Hospital, Hallym University College of Medicine, Hwaseong-si 18450, Republic of Korea; konanlover@hanmail.net (E.J.K.); jrkoomd@gmail.com (J.-R.K.); 2 Hallym Kidney Research Institute, Hallym University, Seoul 07441, Republic of Korea; ajincho@hallym.or.kr (A.C.); dhkim6489@hanmail.net (D.H.K.); unionbey1@hallym.or.kr (J.Y.Y.); nnssl@hallym.or.kr (K.S.); ejej9377@hallym.or.kr (E.K.); 3 Department of Internal Medicine, Graduate School of Kangwon National University, Kangwon 24341, Republic of Korea; 4 Department of Internal Medicine, Kangnam Sacred Heart Hospital, Hallym University College of Medicine, Seoul 07441, Republic of Korea

**Keywords:** chronic kidney disease, diabetes mellitus, geriatric nutritional risk index, glomerular filtration rate

## Abstract

The aim of this study was to evaluate whether the geriatric nutritional risk index (GNRI) is associated with chronic kidney disease (CKD) progression in patients with type 2 diabetes mellitus (DM). In total, 1100 patients with type 2 DM with a follow-up duration > 1 year were included in this longitudinal study. The risk of CKD progression was assessed according to GNRI quartiles. Patients in the lowest GNRI quartile exhibited a significantly lower estimated glomerular filtration rate (eGFR), compared with those in quartile four. Moreover, these patients had poorer glycemic control and lower hemoglobin levels, body mass index, and albumin levels. Additionally, they exhibited a greater annual decline in eGFR. Multivariate logistic regression analysis showed that old age (>60 years), baseline eGFR, the presence of proteinuria, the use of angiotensin-converting enzyme inhibitors or angiotensin receptor blockers, and low GNRI were significantly associated with CKD progression. GNRI may serve as a valuable predictive tool for identifying the risk of adverse renal outcomes in patients with type 2 DM. It may potentially serve as a more feasible measure for assessing the nutritional status of these patients, as well as for predicting their clinical outcomes.

## 1. Introduction

Chronic kidney disease (CKD) is an important and common public health problem affecting 8–16% of adults worldwide. Nutrition plays an important role in the clinical outcomes of patients with CKD [1,2]. Moreover, malnutrition is very common and associated with adverse clinical outcomes in these patients [1,3,4,5]. Uremic toxin accumulation, metabolic acidosis, intestinal dysbiosis, systemic inflammation, anabolic hormone resistance, and elevated protein catabolism contribute to malnutrition in patients with CKD [3]. In addition, CKD patients with diabetes mellitus (DM) have a higher incidence of protein-energy wasting than those without DM [6]. Therefore, the Kidney Disease Outcomes Quality Initiative (KDOQI) 2020 nutrition guidelines recommend that patients with DM and CKD should maintain a protein intake of 0.8 g/kg/day [7].

Limited tools are available for the assessment of nutritional status. The most commonly used tool for nutritional assessment is the subjective global assessment (SGA), which includes medical history (weight loss, dietary intake, gastrointestinal symptoms and functional capacity) and physical examination (subcutaneous fat, muscle wasting, edema, and ascites). Using the SGA, approximately 20–50% of patients have been reported to be malnourished or at a high risk of malnutrition upon hospital admission [8]. Several nutritional assessment tools have been identified for evaluating nutritional risk, including the Mini Nutritional Assessment Short-Form, mid-arm muscle area, body mass index (BMI), bioelectrical impedance analysis, and geriatric nutritional risk index (GNRI) [8]. However, a universally accepted gold standard or preferred method for evaluating nutritional status has yet to be established [8]. Among these tools, the GNRI is frequently used for nutritional assessment because of its simplicity in clinical practice. Compared with the SGA, the GNRI was originally designed to assess the nutritional status of hospitalized elderly patients and as an objective tool to evaluate parameters including height, body weight, and serum albumin concentration [9]. In previous studies, GNRI was strongly related to mortality in patients undergoing hemodialysis [10]. A low GNRI score was independently associated with end-stage kidney disease progression [11].

Protein-energy wasting is prevalent in patients with CKD and is linked to unfavorable clinical consequences, including high hospitalization and mortality rates [12,13] Moreover, CKD patients with DM have a greater prevalence of protein-energy wasting compared with CKD patients without DM [14]. However, the association between nutritional markers and renal disease in patients with type 2 DM remains unclear. Therefore, we aimed to evaluate whether GNRI is associated with renal progression in patients with type 2 DM.

## 2. Material and Methods

### 2.1. Study Design and Patients

From August 2006 to February 2014, 1940 patients with type 2 DM were enrolled in this study, all of whom received follow-up care at the Department of Endocrinology of Kangnam Sacred Heart Hospital in Seoul, Korea.

**Figure 1:**
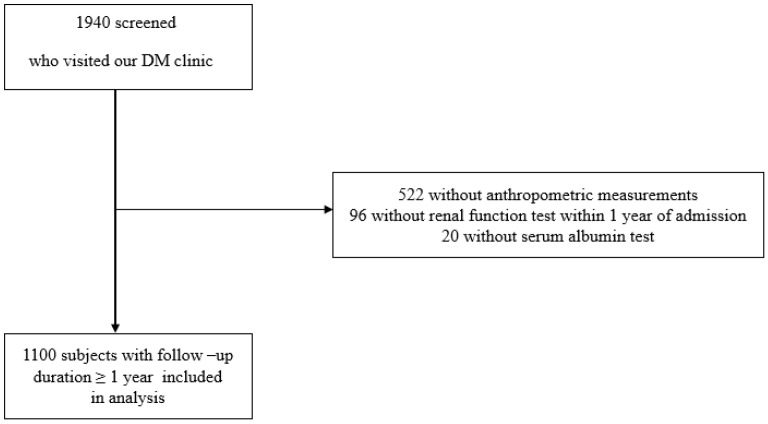
Flow chart of study population. A total of 1940 type 2 DM patients who visited the Department of Endocrinology at Kangnam Sacred Heart Hospital in Seoul, Korea, were screened. Of this group, a total of 522 patients did not undergo anthropometric measurements, 96 patients did not receive a renal function test within 1 year of admission, and 20 patients were unavailable for serum albumin data. Therefore, 1100 patients with follow-up duration over 1 year were included in analysis.

Upon admission, height, weight, and BMI were measured, and a blood test was performed. Ninety-six patients were excluded due to the absence of a renal function examination within 1 year of admission. Additionally, 522 patients without anthropometric measurements and 20 patients without serum albumin measurements were excluded. Due to loss of follow up for a duration exceeding one year, 202 patients were further excluded from the study. Consequently, this longitudinal study included only 1100 patients with a follow-up duration ≥ 1 year (Figure 1). This research was conducted in compliance with the Declaration of Helsinki and received approval from the Institutional Review Board of Hallym University, Kangnam Sacred Heart Hospital (IRB No: 2018-01-030). The requirement of written informed consent from the patients was exempted by the Institutional Review Board.

### 2.2. GNRI Calculation

The GNRI is a modified nutritional risk index. The GNRI was regarded as a simplistic method for assessing nutritional status. The method included three parameters of serum albumin levels, body weight, and height [15]. The GNRI formula is derived by replacing the ideal weight parameter of the nutritional risk index formula with the usual weight, which is estimated using the Lorentz formulae: height (cm) − 100 − ((height (cm) − 150)/4) for men and height (cm) − 100 − ((height (cm) − 150)/2.5) for women [15].

GNRI calculation: GNRI = (1.489 × albumin [g/L]) + (41.7 [weight/ideal weight])

The patients were divided into four groups according to GNRI quartiles (Q1, Q2, Q3, and Q4).

### 2.3. Definition of CKD Progression and Albuminuria Progression

The progression of CKD was determined by the occurrence of one or more of the following: (1) decline in estimated glomerular filtration rate (eGFR) category (≥90 [G1], 60–89 [G2], 45–59 [G3a], 30–44 [G3b], 15–29 [G4], <15 [G5] mL/min/1.73 m^2^) accompanied by a ≥25% reduction in eGFR from baseline; (2) sustained decline in eGFR of >5 mL/min/1.73 m^2^/year [16]. Albuminuria progression was defined by one or more level of progression in albuminuria: normo-albuminuria (urine albumin-creatinine ratio [UACR] < 30 mg/g to micro-albuminuria UACR 30–300 mg/g) or macro-albuminuria (UACR > 300 mg/g) and micro-albuminuria to macro-albuminuria [17].

### 2.4. Laboratory Data Measurement

Baseline characteristics according to GNRI quartiles were measured, including age; sex; presence of hypertension; duration of DM; hemoglobin, calcium, phosphorus, serum creatinine, total cholesterol, hemoglobin A1c (HbA1C), and albumin levels; eGFR; use of angiotensin-converting enzyme inhibitors (ACEI) or angiotensin receptor blockers (ARB); and UACR. The eGFR was calculated using the Chronic Kidney Disease Epidemiology Collaboration (CKD-EPI) equation.

### 2.5. Statistical Analysis

All statistical analyses were performed using SPSS software (version 20.0; SPSS Inc., Chicago, IL, USA). Statistical significance was set at *p* < 0.05 for all analyses. Descriptive statistics are presented as percentages, mean ± standard deviation or medians (interquartile, IQR). Binary logistic regression analysis was performed to evaluate risk factors for CKD progression. One-way analysis of variance (ANOVA) was performed to study the association between GNRI categories and CKD progression.

Univariate analysis was performed to identify risk factors for CKD progression and rate of eGFR decline. Furthermore, univariate and multivariate logistic regression analyses were performed for all significant risks of CKD progression. A direct comparison between GNRI and CKD progression was performed using multivariate models.

## 3. Results

### 3.1. Baseline Characteristics of the Patients According to GNRI Quartiles

**Figure 2:**
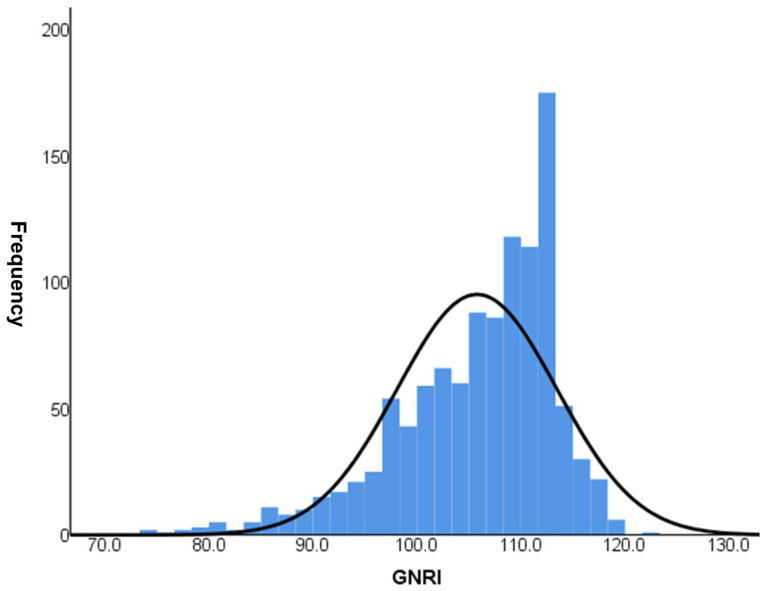
GNRI distribution of the study among 1100 patients.

In total, 1100 patients with type 2 DM (median duration 10.0 [5.0–15.0] years within a median follow up of 9.2 ± 7.7 years) were included. The mean age of the patients was 57.3 ± 11.2 years, 527 (47.9%) patients were male, and 529 (49.7%) patients had hypertension. Patients with CKD stages 1–4 were distributed as follows: 380, 516, 196, and 8, respectively. The distribution of GNRI in the study population is shown in Figure 2. The median GNRI score of the study population was 107.2. Table 1 presents a comparison of the clinical and laboratory parameters according to GNRI quartiles. The median GNRI values for each quartile were 96.8, 105.7, 108.8, and 113.2, respectively. Patients in Q1 had a longer duration of DM and higher levels of fasting plasma glucose and HbA1C than those in the other three quartiles. In addition, serum levels of hemoglobin, calcium, total cholesterol and albumin, and BMI were lower in Q1 than in the other three quartiles. The UACR was higher in Q1 than in the other groups (*p* < 0.001).

### 3.2. Effect of GNRI on the Rate of Renal Function Decline

**Figure 3:**
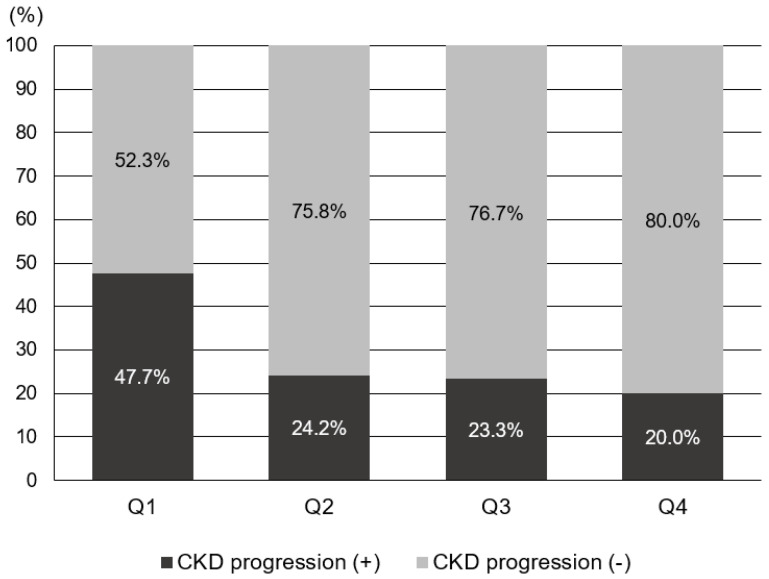
Proportion of the subjects with CKD progression according to GNRI quartile.

**Figure 4:**
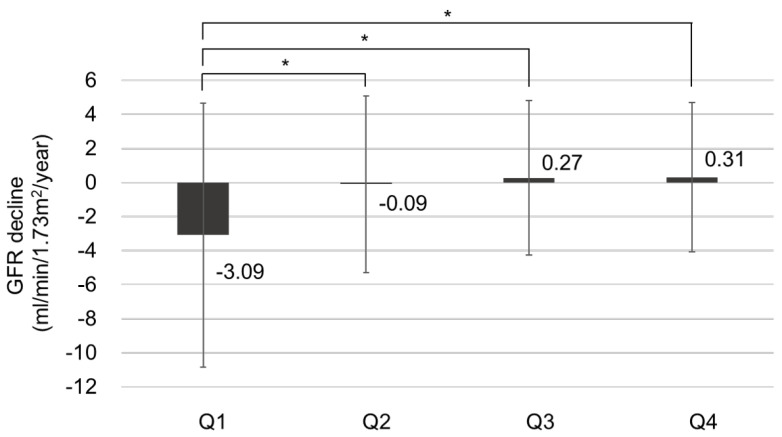
The annual renal function decline rate based on GNRI quartile, * *p* < 0.05.

To evaluate the risk factors for CKD progression, the patients were further divided into CKD progression and non-CKD progression groups (Table 2). Risk factors associated with CKD progression include a longer duration of DM and higher HbA1c levels. Additionally, patients in the CKD progression group demonstrated lower serum hemoglobin and calcium levels, as well as lower serum albumin levels. The CKD progression group also had higher baseline serum creatinine levels, increased albuminuria, lower baseline eGFR, and higher fasting blood glucose levels compared with the non-CKD progression group. The prescription rates of ACEI or ARB were higher in the CKD progression group. The average GNRI score of those with CKD progression was lower than that of those without CKD progression [104.2 (96.8–110.2) vs. 108.7 (103.7–111.7), *p* < 0.001]. The proportion of patients with GNRI Q1 in the CKD progression group was 134 patients (52.3%), significantly higher than that of patients without CKD progression (147 [47.7%]). Annual decline in eGFR was significantly higher in Q1 (−3.088 mL/min/year) than in Q2 and Q3, while it was significantly lower in Q4 (0.310 mL/min/year) than in Q1, according to the statistical analysis (*p* < 0.05). There was no statistically significant difference in annual eGFR decline rates between Q2, Q3, and Q4. The proportion of patients with CKD progression according to GNRI quartiles is shown in Figure 3. In Q1, the proportion of patients with CKD progression was significantly higher (47.7%) than those in the other quartiles (*p* < 0.05). Moreover, annual eGFR decline rates based on GNRI quartile are presented in Figure 4. In the first quartile, patients exhibited a significantly accelerated decline in renal function compared to the second, third, and fourth quartiles (−3.09 ± 7.75 vs. −0.09 ± 5.19, 0.27 ± 4.53, and 0.31 ± 4.38 mL/min/1.73 m^2^ per year, respectively; *p* < 0.001).

### 3.3. GNRI as a Risk Factor for CKD Progression

To assess whether the GNRI is a prognostic factor for CKD progression, we performed a logistic regression analysis (Table 3). In the univariate analysis, DM duration, HbA1C, serum calcium level, low hemoglobin concentration (<10.0 g/dL), baseline eGFR, presence of proteinuria, use of ACEI or ARB, and the lowest quartile (Q1) were related to CKD progression. In the multivariate analysis, old age (age > 60 years) (odds ratio [OR] = 2.320, 95% confidence interval [CI] 1.312–4.100, *p* = 0.004), baseline eGFR (OR = 1.025, 95% CI 1.010–1.040, *p* = 0.001), the presence of proteinuria (OR = 20.311, 95% CI 6.904–59,750 *p* < 0.001), use of ACEI or ARB (OR = 2.004, 95% CI 1.171–3.428, *p* = 0.011), and Q1 (OR = 2.526, 95% CI 1.156–5.521, *p* = 0.020) were significantly associated with CKD progression. We excluded albumin level from the multivariate analysis because the GNRI already includes albumin as a component.

## 4. Discussion

In this study, we investigated the association between nutritional status and CKD progression in patients with type 2 DM. Our findings demonstrated that a low GNRI score is a significant prognostic indicator for CKD progression. Additionally, patients with lower GNRI scores had poor glycemic control and decreased hemoglobin levels, as well as low BMI and albumin levels.

Malnutrition is an extremely important risk factor for both morbidity and mortality and is common in both developed and developing countries [18]. While malnutrition in developing countries is associated with poor socioeconomic conditions, malnutrition in developed countries typically appears in the context of acute or chronic disease [19,20]. CKD is notably linked to protein–calorie malnutrition [21]. In one study, protein-energy wasting was prevalent in 31% of adult CKD patients as assessed using SGA [22].

Serum albumin level and BMI are also used as markers of nutritional status. However, they may be insufficient due to the influence of several factors, including proteinuria, fluid status, and inflammation [15,23].

Furthermore, the relationship between BMI and renal function deterioration among individuals with CKD stages is well-established. Increased BMI (overweight and obesity) is associated with a poor renal outcome [24]. However, some studies have demonstrated that there is no association between BMI and renal outcome [25], while others have reported a significant relationship between BMI and renal outcome only in males [26,27]. Notably, BMI does not differentiate between muscle and fat mass; as a result, an individual with an elevated muscle mass but normal fat mass can be misdiagnosed as an obese patient on BMI alone [26]. In contrast, the GNRI is a good tool for evaluating and predicting the nutritional status of patients reflecting two components [15]. Low GNRI values are mainly influenced by malnutrition or protein-energy wasting.

The GNRI was initially developed for the purpose of predicting malnutrition-related complications and mortality in elderly patients during their hospitalization [15]. However, some studies have shown the inclusion of younger patients in the application of GNRI. Liu et al. demonstrated the effectiveness of GNRI as a valuable screening tool for identifying a high risk of malnutrition among acutely injured trauma patients, including both elderly and young adults [28]. The GNRI has recently been demonstrated to be a simple and objective tool for assessing nutritional status in various pathological conditions [29,30]. Therefore, our study used the GNRI as a nutritional assessment tool which is simpler than several other nutritional screening measures [30].

Previous studies have shown that GNRI is associated with all-cause and cardiovascular mortality in hemodialysis patients [31]. However, whether the GNRI affects renal outcomes in patients with CKD is controversial. Kou et al. reported that low GNRI was independently associated with renal progression to dialysis in patients with advanced CKD [11]. However, Kiuchi et al. showed that a lower GNRI in patients with CKD was significantly associated with mortality and cardiovascular events but had no effect on renal outcomes, despite large amounts of proteinuria [23]. In addition, the association between GNRI and CKD-progression patients with type 2 DM remains unclear. Few studies have evaluated renal outcomes according to GNRI groups in patients with type 2 DM.

Our study found that the lowest GNRI quartile (Q1) was significantly associated with CKD progression. In Q1, the proportion of type 2 DM patients with CKD progression was 47.7%. Our analysis showed that patients in the lowest GNRI quartile had a significantly a higher risk of CKD progression than those in the highest quartile. The main goal of diabetic nephropathy treatment is to slow the progression of renal dysfunction by preventing the progression of microalbuminuria to proteinuria. In patients with type 2 DM, the UK prospective diabetes study reported microalbuminuria and reduced eGFR in 38% and 29% of patients, respectively, after a median follow-up of 15 years [32,33]. Traditionally, the risk factors for diabetic nephropathy include family history, high blood pressure, dyslipidemia, high HbA1C, proteinuria, and smoking [32,34,35]. Therefore, treatment focuses on glycemic control, the use of antihypertensive drugs such as ARB or ACEI, and dyslipidemia improvement.

However, in our opinion, assessing and recognizing nutritional status is imperative in patients with type 2 DM. Patient nutritional status is a modifiable factor that may influence diabetic nephropathy processes and renal outcomes [36,37]. Thus, considering our findings and those from the aforementioned studies, we can conclude that GNRI, calculated using both serum albumin level and weight, may be useful in clinical practice as an objective and inexpensive nutritional marker for monitoring CKD progression in patients with type 2 DM.

Furthermore, this study indirectly suggests the importance of preventing protein-energy wasting and managing its progression in patients with CKD. The prevalence of malnutrition continues to be high, contributing significantly to a multitude of consequential issues in CKD [38]. The International Society of Renal Nutrition and Metabolism (ISRNM) presented the etiological factors for protein-energy wasting. The development of protein-energy wasting is attributed to several factors, including anorexia, declining kidney function, the presence of uremic toxins, and various metabolic abnormalities and comorbidities such as DM, cardiovascular disease, and depression [21,39]. Eventually, protein-energy wasting is one of the intrinsic components of the natural course of CKD [39].

For patients with non-dialysis-dependent CKD, the KDOQI guidelines suggest a recommended protein intake of 0.6–0.8 g/kg body weight per day, particularly for patients with diabetes [7]. The European Society for Clinical Nutrition and Metabolism (ESPEN) guidelines recommend 0.55–0.60 g/kg body weight per day or 0.28 g/kg body weight per day with essential amino acid [40]. The recommendations for protein intake and energy intake in non-dialysis-dependent CKD have not been defined in the European Best Practice Guidelines [41]. However, in non-dialysis-dependent CKD, both KDOQI and ESPEN guidelines recommend approximately 25–35 kcal/kg body weight per day, and are quite similar [7,40,42].

When following protein-restricted diet guidelines like these, it is essential to maintain an adequate dietary energy intake to prevent protein breakdown and the development of protein-energy wasting. Campbell et al. demonstrated that nutrition counseling resulted in enhanced nutritional intake, improved serum albumin levels, and better quality of life [43]. Therefore, by utilizing the nutritional marker known as GNRI, nutritional counseling can help prevent the progression of CKD. Further prospective studies are needed to determine if the improvement of nutritional status in CKD patients is related to renal outcomes.

However, this study has several limitations. First, this was a single-center study, and the data collected retrospectively were limited to the Korean population. Due to the specific population and conditions of our study, the generalizability of our findings may be limited. Second, we did not collect 24 h urine samples to measure the albumin-to-creatinine ratio. Furthermore, the study data of some patients were lost during serial follow-up of albuminuria. Fourth, we did not adjust for important factors that could potentially impact CKD progression, including inflammatory markers such as C-reactive protein, dietary patterns, unhealthy lifestyle (smoking or drinking), markers of vascular stiffness or cardiac arrythmia, and metabolic factors like FGF23. Finally, this study design cannot be applied to the general population, regardless of race. Despite these limitations, this study has several strengths. To our knowledge, our study is the first to examine the relationship between the GNRI and CKD progression in patients with type 2 DM. In addition, this study included a relatively large number of patients with type 2 DM and a prolonged follow-up period. Further prospective cohort-based studies are warranted to validate the GNRI.

## 5. Conclusions

In conclusion, the GNRI may be an effective tool for predicting CKD progression in patients with type 2 DM. Clinicians should be aware of the significance of nutritional status in CKD progression, particularly in patients with type 2 DM.

## Figures and Tables

**Table 1:** Baseline characteristics of the study population according to the GNRI category.

Variables	Total (*n* = 1100)	Q1 (*n* = 281)	Q2 (*n* = 298)	Q3 (*n* = 236)	Q4 (*n* = 285)	*p*-Value
Age (years)	57.3 ± 11.2	57.5 ± 12.6	59.0 ± 10.6	57.2 ± 10.1	55.6 ± 11.1	0.005
Male (%)	527 (47.9)	132 (47.0)	134 (45.0)	107 (45.3)	154 (54.0)	0.11
Hypertension (%)	529 (49.7)	131 (47.3)	151 (52.1)	117 (51.8)	130 (47.8)	0.557
SBP (mmHg)	130.0 (118.0–140.0)	130.0 (112.0–140.0)	130.0 (120.0–130.0)	130.0 (117.0–140.0)	123.5 (116.3–138.5)	0.208
DBP (mmHg)	79.0 (70.0–85.0)	80.0 (70.0–90.0)	80.0 (70.0–87.0)	78.0 (70.0–85.0)	74.0 (68.0–80.0)	0.096
DM duration (years)	10.0 (5.0–15.0)	11.0 (5.0–17.0)	10.0 (5.0–15.0)	9.0 (4.0–15.0)	9.0 (5.0–14.0)	0.016
HbA1c (%)	7.3 (6.5–78.9)	8.3 (6.78–10.2)	7.1 (6.5–8.5)	7.0 (6.4–8.2)	7.2 (6.5–8.0)	<0.001
FPG (mg/dL)	134.0 (109.0–177.0)	148.0 (108.5–209.0)	131.0 (110.0–174.5)	128.5 (108.0–166.3)	131.0 (108.0–169.0)	<0.001
ACEI or ARB (%)	517 (47.0)	137 (48.8)	138 (46.3)	106 (44.9)	136 (47.7)	0.831
Hemoglobin (g/dL)	7.3 (6.5–8.9)	11.9 (10.6–13.4)	13.2 (12.2–14.4)	13.7 (12.7–14.7)	13.8 (12.6–14.9)	<0.001
Creatinine (mg/dL)	0.91 (0.78–1.1)	0.95 (0.78–1.15)	0.89 (0.78–1.1)	0.91 (0.77–1.08)	0.91 (0.78–1.1)	0.646
eGFR (mL/min/1.73 m^2^)	80.0 (66.0–95.3)	77.5 (61.2–95.4)	80.0 (65.9–96.0)	80.4 (66.3–94.5)	82.7 (68.7–96.0)	0.114
Calcium (mg/dL)	8.9 (8.6–9.2)	8.4 (8.0–8.7)	8.9 (8.6–9.1)	9.0 (8.8–9.4)	9.2 (9.0–9.5)	<0.001
Phosphorus (mg/dL)	3.6 (3.2–4.0)	3.6 (3.0–4.1)	3.6 (3.2–4.0)	3.6 (3.3–4.0)	3.7 (3.3–4.0)	0.062
Total cholesterol (mg/dL)	162.0 (140.0–189.0)	153.0 (129.0–183.0)	163.0 (141.0–186.0)	165.0 (142.0–195.0)	166.0 (146.0–191.0)	0.011
Albumin (g/dL)	4.4 (4.1–4.7)	3.8 (3.5–4.0)	4.3 (4.2–4.4)	4.6 (4.5–4.6)	4.8 (4.7–4.9)	<0.001
UACR (mg/g)	15.2 (8.2–36.0)	22.5 (9.3–114.4)	16.3 (9.4–41.6)	13.4 (7.0–25.2)	13.8 (7.9–26.0)	<0.001
BMI (kg/m^2^)	24.7 (22.5–27.1)	23.6 (20.8–26.2)	25.1 (22.4–27.4)	24.8 (22.8–27.4)	25.2 (23.4–27.2)	<0.001
GNRI	107.2 (101.3–111.7)	96.8 (92.3–99.8)	105.7 (104.2–107.2)	108.8 (108.7–110.2)	113.2 (111.7–114.7)	<0.001

Data are expressed as mean ± standard deviation, or medians (interquartile range), and number (percent). Abbreviations: ACEI, angiotensin-converting enzyme inhibitor; BMI, body mass index; DBP, diastolic blood pressure; eGFR, estimated glomerular filtration rate; FPG, fasting plasma glucose; GNRI, geriatric nutritional risk index; HbA1c, hemoglobin A1c; SBP, systolic blood pressure; UACR, urine albumin–creatinine ratio.

**Table 2:** Risk factors associated with CKD progression.

Parameters	CKD Progression		*p* Value
	(−) (*n* = 782)	(+) (*n* = 318)	
Age (years)	57.1 ± 11.1	58.0 ± 11.4	0.216
Male (%)	363 (46.4%)	164 (51.6%)	0.121
Hypertension (%)	365 (48.5%)	164 (52.4%)	0.251
SBP	130.0 (118.0–140.0)	130.0 (118.0–140.0)	0.328
DBP	79.0 (70.0–83.8)	80.0 (70.0–87.0)	0.921
DM duration (years)	9.0 (4.0–14.0)	13.0 (6.0–18.0)	<0.001
HbA1c (%)	7.1 (6.5–8.4)	7.9 (6.7–9.7)	<0.001
FPG (mg/dL)	131.5 (109.0–170.0)	144.0 (110.0–202.0)	0.006
ACEI or ARB (%)	348 (44.5)	169 (53.1)	0.009
Hemoglobin (g/dL)	13.4 (12.4–14.4)	12.3 (11.0–14.1)	<0.001
Creatinine at baseline (mg/dL)	0.9 (0.77–1.07)	0.94 (0.8–1.2)	0.004
eGFR at baseline(mL/min/1.73 m^2^)	80.7 (69.0–95.0)	75.7 (59.6–95.4)	0.006
Calcium	9.0 (8.6–9.3)	8.8 (8.3–9.2)	<0.001
Phosphorus	3.6 (3.2–4.1)	3.6 (3.2–4.0)	0.082
Total cholesterol (mg/dL)	163.0 (141.0–189.0)	162.0 (138.0–191.0)	0.934
Albumin (g/dL)	4.5 (4.2–4.7)	4.2 (3.8–4.6)	<0.001
UACR at baseline (mg/g)	13.7 (7.5–27.1)	22.6 (10.2–129.2)	0.004
BMI (kg/m^2^)	24.7 (22.5–27.0)	24.8 (22.5–27.3)	0.327
GNRI score	108.7 (103.7–111.7)	104.2 (96.8–110.2)	<0.001
GNRI Q1	147 (18.8)	134 (42.1)	<0.001

Data are expressed as mean ± standard deviation, or medians (interquartile range), and number (percent). Abbreviations: ACEI, angiotensin-converting enzyme inhibitor; BMI, body mass index; DBP, diastolic blood pressure; eGFR, estimated glomerular filtration rate; FPG, fasting plasma glucose; GNRI, geriatric nutritional risk index; HbA1c, hemoglobin A1c; SBP, systolic blood pressure; UACR, urine albumin–creatinine ratio.

**Table 3:** Logistic regression analysis for the risk of CKD progression.

Variables	Univariate		Multivariate	
	OR (95% CI)	*p* Value	OR (95% CI)	*p* Value
Age > 60	1.199 (0.921–1.559)	0.177	2.320 (1.312–4.100)	0.004
Female gender	1.229 (0.947–1.596)	0.121	1.610 (00966–2.686)	0.068
DM duration ≥ 10 years	2.041 (1.561–2.669)	<0.001	1.482 (0.780–2.523)	0.148
HbA1c ≥ 7.0%	1.651 (1.247–2.186)	<0.001	0.986 (0.587–1.674)	0.957
Calcium	0.570 (0.450–0.721)	<0.001	1.543 (0.864–2.754)	0.142
Phosphorus	0.822 (0.667–1.014)	0.068		
Total cholesterol (mg/dL)	1.00 (0.997–1.003)	0.928		
Hemoglobin < 10 g/dL	3.101 (1.955–4.920)	<0.001	1.540 (0.424–5.595)	0.512
SBP	1.004 (0.996–1.012)	0.328		
DBP	1.001 (0.998–1.013)	0.921		
Baseline eGFR (mL/min/1.73 m^2^)	0.991 (0.985–0.997)	0.003	1.025 (1.010–1.040)	0.001
Presence of proteinuria	15.958 (7.125–35.744)	<0.001	20.311 (6.904–59.750)	<0.001
Use of ACEI or ARB	1.781 (1.424–2.153)	<0.001	2.004 (1.171–3.428)	0.011
GNRI Q1 vs. Q2,3,4	3.146 (2.364–4.187)	<0.001	2.526 (1.156–5.521)	0.020

Abbreviations: ACEI, angiotensin-converting enzyme inhibitor; CKD, chronic kidney disease; CI, confidence interval; DBP, diastolic blood pressure; eGFR, estimated glomerular filtration rate; GNRI, geriatric nutritional risk index; HbA1c, hemoglobin A1c; OR, odds ratio; SBP, systolic blood pressure.

## Data Availability

The authors confirm that the data supporting the findings of this study are available.
